# Sensitive Hg^2+^ Sensing via Quenching the Fluorescence of the Complex between Polythymine and 5,10,15,20-tetrakis(*N*-methyl-4-pyridyl) Porphyrin (TMPyP)

**DOI:** 10.3390/s18113998

**Published:** 2018-11-16

**Authors:** Daohong Wu, Yaliang Huang, Shengqiang Hu, Xinyao Yi, Jianxiu Wang

**Affiliations:** College of Chemistry and Chemical Engineering, Central South University, Changsha 410083, China; wudaohong@csu.edu.cn (D.W.); 182301010@csu.edu.cn (Y.H.); zn_sqhu@csu.edu.cn (S.H.); jxiuwang@csu.edu.cn (J.W.)

**Keywords:** interaction, polythymine, TMPyP, Hg^2+^, synergistic effect, ternary complex

## Abstract

The interaction between polythymine (dTn) and 5,10,15,20-tetrakis(N-methyl-4-pyridyl) porphyrin (TMPyP) was systematically studied using various techniques. dTn remarkably enhanced the fluorescence intensity of TMPyP as compared to other oligonucleotides. The enhanced fluorescence intensity and the shift of the emission peaks were ascribed to the formation of a *π*-*π* complex between TMPyP and dTn. And the quenching of the dTn-enhanced fluorescence by Hg^2+^ through a synergistic effect occurs due to the heavy atom effect. The binding of Hg^2+^ to TMPyP plays an important role in the Hg-TMPyP-dT_30_ ternary complex formation. A TMPyP-dT_30_-based Hg^2+^ sensor was developed with a dynamic range of Hg^2+^ from 5 nM to 100 nM. The detection limit of 1.3 nM was low enough for Hg^2+^ determination. The sensor also exhibited good selectivity against other metal ions. Experiments for tap water and river water demonstrated that the detection method was applicable for Hg^2+^ determination in real samples. The Hg^2+^ sensor based on oligonucleotide dT_30_-enhanced TMPyP fluorescence was fast and low-cost, presenting a promising platform for practical Hg^2+^ determination.

## 1. Introduction

As one of the most important conjugated organic molecules, porphyrins play a crucial role in the metabolism of living organisms [1]. For example, porphyrins are responsible for the production of singlet oxygen, which could damage DNA in tumor cells [2]. The interaction between porphyrins or their derivatives and nucleic acids has been extensively investigated [3,4,5], in which three binding types including intercalation, external or groove binding, and outside stacking were involved [6].

5,10,15,20-tetrakis(N-methyl-4-pyridyl)porphyrin (TMPyP) is a water-soluble cationic porphyrin, which contains a porphyrin core and N-methylpyridinium side chains [7]. Due to their planarity and hydrophobicity, the porphyrin rings can intercalate into the base pairs of DNA and in the same time, the positively charged side chains can electrostatically interact with the negatively charged nucleic acids [8]. For example, evidenced by nuclear magnetic resonance spectroscopy, TMPyP can intercalate into GC-rich regions of duplex DNA [9,10]. However, the binding of TMPyP to the major groove of the AT-rich regions has also been proposed [11]. TMPyP has been reported to interact with triplex DNA, in which the third strand could inhibit the assembly of TMPyP with duplex DNA [12]. TMPyP can also bind to the G-quadruplex structure through external stacking rather than intercalation between the G-tetrads [13,14,15]. As probably the most flexible form, the single stranded DNA enables unrivaled access to individual bases [6], which facilitates the interaction with TMPyP. 

**Figure 1:**
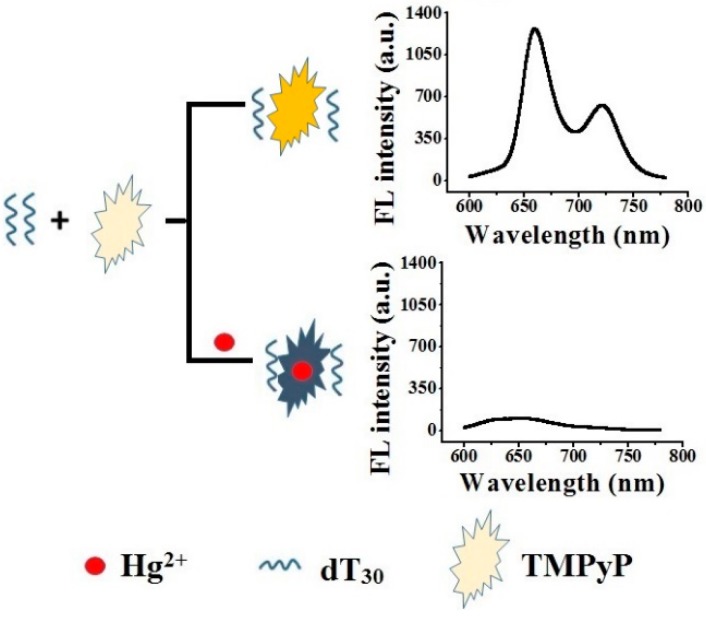
Schematic of Hg^2+^ assay by quenching the fluorescence of TMPyP-dT_30_.

Mercuric ion (Hg^2+^) possesses serious immunotoxic, genotoxic, and neurotoxic effects, causing damage to the central nervous system, endocrine system and other organs such as the kidney, liver, heart, and lung [16]. Hg^2+^ usually serves as a fluorescent quencher through enhancement of spin-orbit coupling [17]. A colorimetric method for Hg^2+^ determination through platinum nanoparticles with limit of detection 8.5 pM and a linear range from 0.01 to 4 nM has been reported by Aragay et al. [18]. MoS_2_ nanosheet/DNA/carbon dot-based fluorescence method has been reported to detect Hg^2+^ [19]. However, most of the methods were based on synthesized nanometer materials such as nanoparticle, quantum dots, nanosheet, which were hard to be modified. Herein, the interaction between TMPyP and polyadenine (dA_n_)_,_ polycytosine (dC_n_)_,_ polyguanine (dG_n_) and polythymine (dT_n_) were investigated. dT_n_ was found to substantially enhance the fluorescence intensity of TMPyP due to the formation of a TMPyP-dT_n_ complex. The incorporation of Hg^2+^ significantly quenched the fluorescence of the complex and a fluorescent sensor for Hg^2+^ was developed. The sensing strategy is shown in Figure 1.

## 2. Materials and Methods

### 2.1. Reagents and Chemicals

5,10,15,20-tetrakis(*N*-methyl-4-pyridyl)porphyrin (TMPyP) was purchased from Sigma-Aldrich (St. Louis, MO, USA). The oligonucleotides (ODNs), such as polyadenine dA_13_, polycytosine dC_13_, polyguanine dG_6_TG_6_ and polythymine dT_5_, dT_10_, dT_13_, dT_15_, dT_20_, dT_30_, dT_40_, dT_50_ were synthesized by Sangon Biotechnology Co., Ltd. (Shanghai, China). Hg(NO_3_)_2_, Cd(NO_3_)_2_·4H_2_O, Cr(NO_3_)_3_·9H_2_O, Cu(NO_3_)_2_·3H_2_O, Fe(NO_3_)_3_·9H_2_O, FeSO_4_·7H_2_O, Mn(CH_3_COO)_2_·4H_2_O, MgCl_2_·6H_2_O, NiCl_2_·6H_2_O, Pb(NO_3_)_2_, ZnCl_2_, Tris-HCl, CH_3_COOH, NaCl and NaOH were acquired from Sinopharm Chemical Reagent Co., Ltd. (Shanghai, China). All the ODNs were prepared with Tris-HCl buffer (10 mM Tris, 100 mM NaCl, pH 7.4). All reagents were of analytical grade and used as received. Ultrapure water was obtained from a Millipore water purification system (≥18 MΩ, Milli-Q, Millipore).

### 2.2. Apparatus

UV-vis spectra and circular dichroism (CD) spectra were recorded on a UV-2450 spectrophotometer (Shimadzu, Japan) and a Jasco-815 spectropolarimeter (Jasco, Japan), respectively. Fluorescence spectra were collected on a Hitachi F-4600 spectrofluorometer (Hitachi, Japan). Fluorescence lifetime was measured on a compact FluoTime 100 fluorescence lifetime spectrometer (PicoQuant GmbH, Germany).

### 2.3. Procedure

The TMPyP-ODN complex was formed by mixing 1 μM TMPyP and 2 μM ODN, followed by dilution to 200 μL with Tris-HCl buffer and stirring for 50 min. For the Hg^2+^ assay, Hg^2+^ with various concentrations was added into the solution of TMPyP-dT_30_ complex under stirring for 1 min. For the fluorescence, measurement with excitation was 422 nm and emission was at 660 nm. The fluorescence lifetimes were determined from the data obtained from a compact FluoTime 100 fluorescence lifetime spectrometer. The data could be analyzed by exponential fits. The solution of Hg^2+^, TMPyP and dT_30_ were mixed by 1 μM Hg^2+^ and 1 μM TMPyP for Hg-TMPyP; 1 μM TMPyP and 2 μM dT_30_ for TMPyP-dT_30_; 1 μM Hg^2+^, 1 μM TMPyP and 2 μM dT_30_ for Hg-TMPyP-dT_30._ The solutions were followed by dilution to 200 μL with Tris-HCl buffer and stirring for 120 min. All the lifetime measurements were with an excitation at 422 nm.

## 3. Results and Discussion

### 3.1. Interaction between ODNs and TMPyP

**Figure 2:**
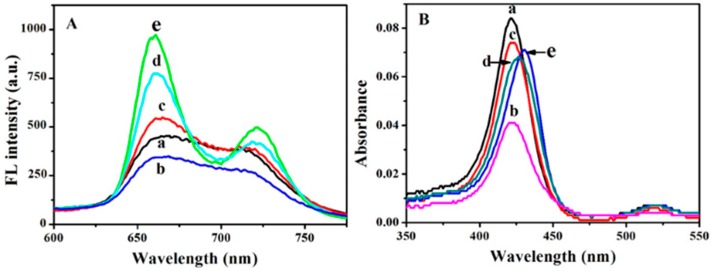
(**A**) Fluorescence and (**B**) UV-vis absorption spectra of 1.0 μM TMPyP in the (a) absence and (b) presence of 2.0 μM dG_6_TG_6_, (c) dA_13_, (d) dC_13_ or (e) dT_13_.

The interaction between TMPyP and ODNs was characterized by fluorescence and UV-vis absorption spectroscopy. As shown in Figure 2A, upon excitation at 422 nm, TMPyP exhibited two broad peaks at around 665 nm and 715 nm (curve a). With the addition of dG_6_TG_6_, the fluorescence of TMPyP decreased due to the electron transfer from guanine to TMPyP (curve b) [20]. However, the incorporation of dA_13_ (curve c), dC_13_ (curve d) and dT_13_ (curve e) increased the fluorescence of TMPyP, and the emission wavelengths were shifted to 660 nm and 720 nm. The enhanced fluorescence intensity and the shift of the emission peaks were ascribed to the formation of a *π*-*π* complex between TMPyP and ODNs [20]. The above results were consistent with those in the cases of mononucleotides. Mononucleotides interacted differently with TMPyP, in which adenine, thymine, and cytosine increased the fluorescence intensity of TMPyP, while guanine substantially quenched the fluorescence intensity of TMPyP [20]. The higher fluorescence of TMPyP by dT_13_ than that by dA_13_ or dC_13_ indicated that TMPyP binded to dT_13_ with stronger affinity [21]. 

TMPyP displayed a characteristic absorption peak at 422 nm known as the Soret band (curve a in Figure 2B), which originated from the S_0_**–**S_2_ transition [22]. The incorporation of dG_6_TG_6_ (curve b), dA_13_ (curve c), dC_13_ (curve d) and dT_13_ (curve e) decreased the absorption of TMPyP, and the absorbance peak of TMPyP was red-shifted to 426 nm and 431 nm in the case of dC_13_ (curve d) and dT_13_ (curve e), respectively. The larger bathochromic shift in curve e suggested the higher binding affinity between TMPyP and dT_13_.

**Figure 3:**
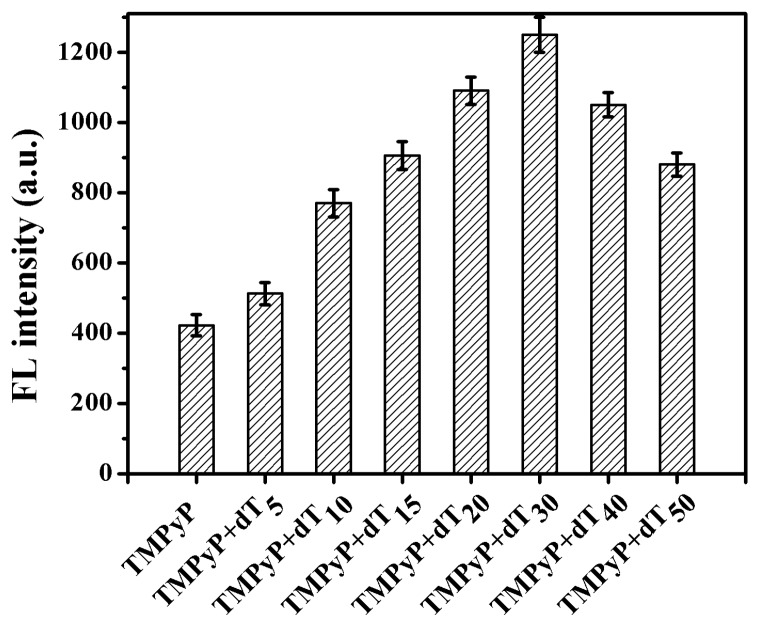
Dependence of fluorescence intensity on the sequence length of poly T. The fluorescence was excited at 422 nm and measured at 660 nm. The absolute errors deduced from three replicate measurements are shown as the error bars.

The length of polythymine also influenced the fluorescence enhancement of TMPyP (Figure 3). The incorporation of different lengths of polythymine resulted in increased fluorescence and began to level off with the length of dT_30_. Thus, the length of dT_30_ might have been more favorable for π-π stacking between TMPyP and polythymine.

### 3.2. Fluorescence Quenching of TMPyP-dT_30_ Complex by Hg^2+^

**Figure 4:**
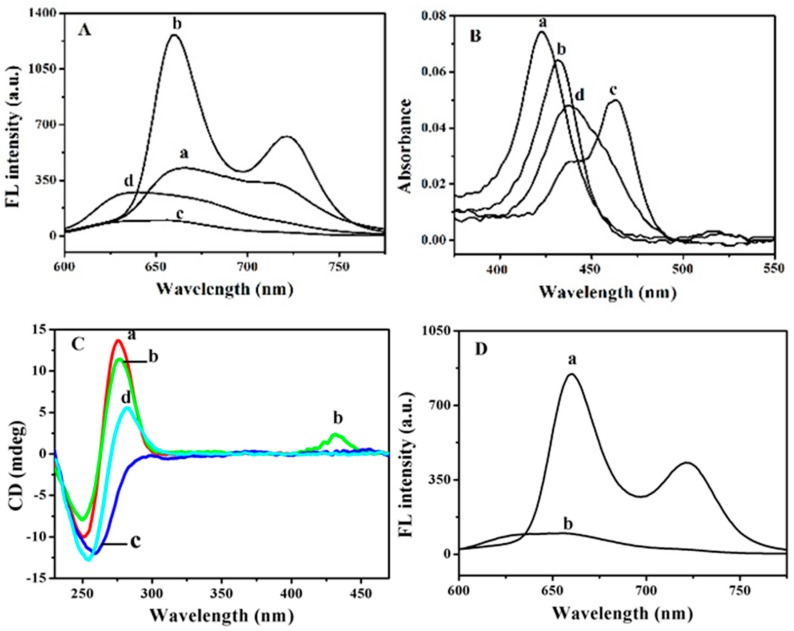
(**A**) Fluorescence and (**B**) UV-vis absorption spectra of (a) 1.0 μM TMPyP, (b) 1.0 μM TMPyP + 2.0 μM dT_30_, (c) 1.0 μM TMPyP + 2.0 μM dT_30_ + 4.0 μM Hg^2+^, (d) 1.0 μM TMPyP + 4.0 μM Hg^2+^. (**C**) CD spectra of (a) 20 μM dT_30_, (b) 20 μM dT_30_ + 100 μM TMPyP, (c) b + 100 μM Hg^2+^, (d) a + 100 μM Hg^2+^. (**D**) Fluorescence spectra of (a) 1.0 μM TMPyP + 2.0 μM dC_30,_ (b) a + 4.0 μM Hg^2+^. The fluorescence was excited at 422 nm and measured at 660 nm.

In comparison with the fluorescence of TMPyP at 665 nm and 715 nm (curve a in Figure 4A), the fluorescence of the TMPyP-dT_30_ complex at 660 nm and 720 nm (curve b in Figure 4A) was significantly enhanced. With the addition of Hg^2+^, the fluorescence of the TMPyP-dT_30_ complex at 660 nm was greatly quenched and in the same time, a tiny and broad emission peak at 635 nm was attained (curve c). The emission peak at 635 nm was ascribed to that of Hg-TMPyP, as evidenced by the emission of Hg-TMPyP in curve d. Porphyrins have been reported to bind to Hg^2+^ with the formation of metalloporphyrin [23], and the fluorescence of porphyrins could be quenched by Hg^2+^ due to the heavy atom effect [24]. Based on the results above, a new ternary complex of Hg-TMPyP-dT_30_ might have formed [25], providing the possibility for a Hg^2+^ assay. 

To further demonstrate the formation of the ternary complex_,_ UV-vis absorption spectra were investigated (Figure 4B). With the addition of dT_30_, the Soret band of TMPyP at 422 nm (curve a) was red-shifted to 431 nm (curve b). The electrostatic interaction and π-π stacking facilitated the formation of the TMPyP-dT_30_ complex [20]. The formation of the ternary complex of Hg-TMPyP-dT_30_ led to a larger bathochromic shift of the Soret band to 462 nm, and in the same time, a new and small peak appeared at 437 nm (curve c). The new peak originated from the absorption of Hg-TMPyP (curve d) [26]. 

The conformational change of dT_30_ induced by TMPyP was studied by CD measurements (Figure 4C). The dT_30_ exhibited a negative peak at 250 nm and a positive peak at 275 nm (curve a). Upon adding TMPyP to the solution of dT_30_, both peaks decreased slightly and in the same time, a new positive peak at 430 nm appeared (curve b), which indicated the binding of TMPyP to dT_30_ through π-π stacking [27,28]. Note that TMPyP did not show any detectable CD signal. With the incorporation of Hg^2+^ to the TMPyP-dT_30_ complex, the 275 nm-peak disappeared and the negative peak at 250 nm shifted to 260 nm (curve c). Furthermore, the 430 nm-peak disappeared. The results above suggested that the binding of TMPyP to dT_30_ was interrupted by Hg^2+^. Such an interruption might have been ascribed to the alteration of the planarity of the TMPyP core induced by Hg^2+^ [5]. When adding Hg^2+^ to dT_30_ solution, the formation of the T–Hg–T complex was characterized by the negative peak at 255 nm and the positive peak at 280 nm (curve d). No positive peak at 280 nm in curve c was shown compared to the T–Hg–T complex, which indicated the different structure of dT_30_ in T–Hg–T and the Hg-TMPyP-dT_30_ complex.

The fluorescence lifetime measurement provided deep insight into the ternary complex formation. The lifetime of TMPyP determined from the decay curve was 4.6 ns, consistent with that reported previously [26]. The lifetime was increased to 10.2 ns upon formation of TMPyP-dT_30_. However, the ternary complex of Hg-TMPyP-dT_30_ possessed a much shorter lifetime of 2.0 ns. Upon adding Hg^2+^ to the solution of TMPyP, a lifetime of 1.2 ns was observed, indicating the binding of Hg^2+^ to TMPyP [26]. 

As reported previously, the binding of TMPyP to dT_30_ possessed high binding affinity (binding constant of 10^7^ M^−1^), while the coordination of Hg^2+^ to dT_30_ was a slow dynamic process at low concentrations of Hg^2+^ [29]. The binding of Hg^2+^ to TMPyP plays an important role in the Hg-TMPyP-dT_30_ ternary complex formation. Upon addition of Hg^2+^ to the solution of TMPyP-dC_30_, the ternary complex of Hg-TMPyP-dC_30_ was also formed, as evidenced by the quenching of the fluorescence of TMPyP-dC_30_ by Hg^2+^ (Figure 4D). Considering the relatively lower affinity between Hg^2+^ and dC_30_ than that between Hg^2+^ and TMPyP [26,30], the coordination of Hg^2+^ to TMPyP was essential for the formation of the Hg-TMPyP-dC_30_ complex. The quenching of the fluorescence of TMPyP-dT_30_ by Hg^2+^ provided the possibility for a Hg^2+^ assay.

### 3.3. Calibration Curve of The Hg^2+^ Assay 

**Figure 5:**
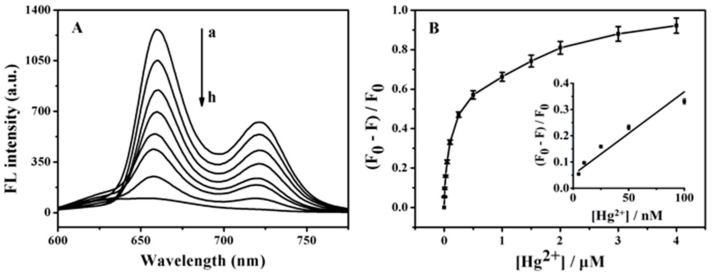
(**A**) Fluorescence spectra of TMPyP-dT_30_ in the presence of Hg^2+^ with various concentrations: 0, 0.025, 0.1, 0.25, 0.5, 1.0, 2.0 and 4.0 μM (from a to h). (**B**) Calibration curve for Hg^2+^ assay. F_0_ and F represent the fluorescence intensities in the absence and presence of Hg^2+^, respectively. The inset shows the linear portion of the curve with Hg^2+^ concentrations ranging from 5 nM to 100 nM. The vertical bars designate the standard deviation for the mean of three replicate measurements.

The fluorescence intensity of TMPyP-dT_30_ at 660 nm gradually decreased with an increased Hg^2+^ concentration (Figure 5A). The dependence of (F_0_ − F)/F_0_ on the concentration of Hg^2+^ was shown in Figure 5B, where F_0_ and F represented the fluorescence intensities in the absence and presence of Hg^2+^, respectively. The inset of Figure 5B depicted the linear portion of the curve with Hg^2+^ concentrations ranging from 5 nM to 100 nM and the linear regression equation was presented as (F_0_ − F)/F_0_ = 3.22 [Hg^2+^] (μM) + 0.05 (*R^2^* = 0.9605). The detection limit was estimated to be 1.3 nM, being much lower than those by the 8-amino BODIPY-based fluorescence assay (49 nM) [31], Raman spectroscopic method (10 nM) [32], colorimetric assay employing plasmonic gold nanoparticles (50 nM [33] and 17.3 nM [34]), colorimetric assay based on Cu_2-x_Se nanoparticles (2.7nM) [35] and the fluorescence polarization assay based on CdTe/CdS quantum dots (8.6 nM) [36]. Such a concentration level was also comparable with those by fluorescent assay based on phosphorothioate RNA modifications (1.7 nM) [37]. The comparison of the proposed sensor with other methods is shown in Table 1. Because the maximum permissible level of Hg^2+^ in drinking water and food, set by the U.S. Environmental Protection Agency and World Health Organization, is at 2 ppb (10 nM) [38], the sensing protocol serves as a viable alternative for a practical Hg^2+^ assay.

### 3.4. Selectivity of the Assay 

**Figure 6:**
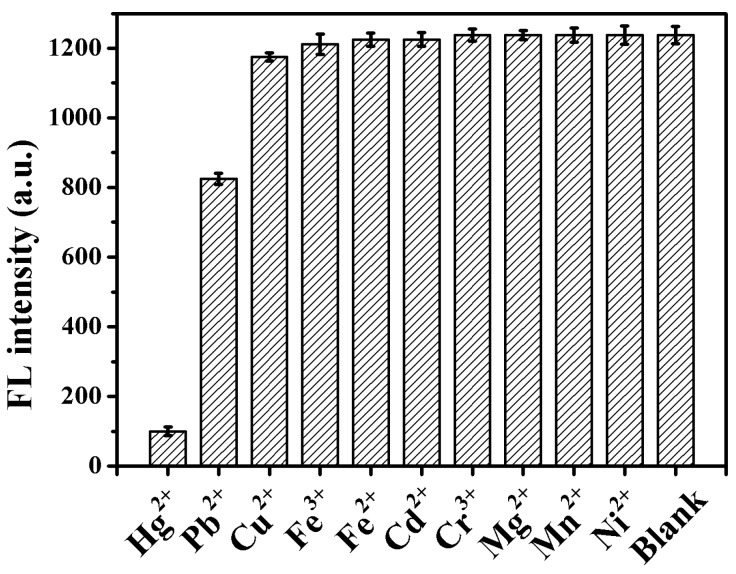
Selectivity of the fluorescence assay of Hg^2+^. The fluorescence intensity of TMPyP-dT_30_ was attained in the absence (Blank) and presence of 4.0 μM of Hg^2+^, Pb^2+^, Cu^2+^, Fe^3+^, Fe^2+^, Cd^2+^, Cr^3+^, Mg^2+^, Mn^2+^ or Ni^2+^. The vertical bars designate the standard deviation of the mean of three replicate measurements.

The selectivity of the method was evaluated by the addition of various metal ions of 4.0 µM (Figure 6). Hg^2+^ quenched about 90% of the fluorescence intensity of the TMPyP-dT_30_ complex at 660 nm. However, other metal ions, such as Cu^2+^, Fe^3+^, Fe^2+^, Cd^2+^, Cr^3+^, Mg^2+^, Mn^2+^ and Ni^2+^ exerted negligible influence on the fluorescence intensity of TMPyP-dT_30_. Pb^2+^ caused 30% quenching of the fluorescence intensity due to the formation of the unstable Pb^2+^-TMPyP-dT_30_ [39]. The proposed fluorescence method possessed good selectivity toward Hg^2+^ determination.

### 3.5. Practical Samples for the Hg^2+^ Assay

The practical application of this method for Hg^2+^ determination was evaluated by the standard addition method through adding different concentration of Hg^2+^ in tap water and river water. As shown in Table 2, the recoveries were between 96–105%. The recovery suggested that the method was largely free from the matrix effect of the complex real water samples and could be served for Hg^2+^ determination in tap water and river water.

## 4. Conclusions

The fluorescence quenching capability of Hg^2+^ on the TMPyP-dT_30_ has been proposed, enabling a sensitive and selective Hg^2+^ assay. The formation of the Hg-TMPyP-dT_30_ ternary complex was characterized by various techniques. The mechanism inherent in the fluorescence quenching of TMPyP-dT_30_ by Hg^2+^ involved a synergistic effect. The detection limit of the proposed method was obtained as 1.3 nM. Experiments for tap water and river water demonstrated that the detection method was applicable for Hg^2+^ determination in real samples. The Hg^2+^ sensor based on oligonucleotide dT_30_-enhanced TMPyP fluorescence was fast and low-cost, presenting a promising platform for practical Hg^2+^ determination. In addition, this is the first time dT_30_ enhanced TMPyP fluorescence intensity was used for Hg^2+^ determination. The sensing protocol may provide useful information on the interaction among porphyrins, DNA and heavy metal ions.

## Figures and Tables

**Table 1:** A comparison of the proposed sensor with other detection methods.

Method	Tool	Linear Range	LOD	Time	Real Sample	Ref.
colorimetric	platinum nanoparticles	0.01–4 nM	0.0085 nM	10 min	tap water	[18]
colorimetric	gold nanoparticles	25–750 nM	50 nM	40 min	pond and river water	[33]
colorimetric	Cu_2-x_Se nanoparticles	0–800 nM	2.7 nM	10 min	tap, pond and river water	[35]
colorimetric	gold nanoparticles and aptamer	10–1000 nM	17.3 nM	20 min	tap, rivers, lakes and ocean water	[34]
Raman spectroscopy	silver nanoparticles	1–1000 μM	10 nM	30 min	drinking mineral water	[32]
fluorescence	Phosphorothioate RNA Modifications	0–50 nM	1.7 nM	20 min	lake water	[37]
fluorescence	MoS_2_ nanosheet/DNA/ carbon dot	0–10 nM	1.02 nM	15 min	tap and lake water	[19]
fluorescence	fluorescent 8-amino BODIPY-based probe	0.5–5 μM	49 nM	50 min	SMMC-7721 cells	[31]
fluorescence polarization	CdTe/CdS QDs	10–800 nM	8.6 nM	2.0 h	lake and spiked lake water	[36]
fluorescence	polyT-TMPyP	5–100 nM	1.3 nM	60 s	Tap and river water	this work

**Table 2:** Determination of Hg^2+^ in water samples with the proposed method.

Sample	Added (nM)	Measured (nM) *^a^*	Recovery (%)
tap water	2.0	2.1 ± 0.17	105 ± 0.085
	5.0	4.9 ± 0.32	98 ± 0.064
	10.0	9.6 ± 0.62	96 ± 0.062
river water	2.0	2.1 ± 0.11	105 ± 0.055
	5.0	5.1 ± 0.43	102 ± 0.086
	10.0	9.7 ± 0.65	97 ± 0.065

*^a^* Mean values and standard deviations were obtained from three independent experiments.
